# An unusual cause of obstructive jaundice: Lemmel’s syndrome

**DOI:** 10.1259/bjrcr.20200166

**Published:** 2020-12-15

**Authors:** Habib Bellamlih, Meryem Echchikhi, Aymane El Farouki, Nabil Moatassim Billah, Ittimade Nassar

**Affiliations:** 1 Department of Radiology, University Hospital Center IBN SINA, Mohammed V-Souissi University, Rabat, Morocco

## Abstract

Lemmel’s syndrome is a rare and misdiagnosed cause of obstructive jaundice. The cause of the obstacle is a duodenal diverticulum located at the periampullary generating a compression effect on the common bile duct with secondary dilation of the extra- and intra-hepatic bile ducts. Late diagnosis of this entity is common and may lead to unnecessary further investigations and therapeutic delay. There are only few case reports of this rare condition. We report a case of 77-year-old female presenting with obstructive jaundice due to Lemmel’s syndrome. The diagnosis was made on a set of clinical, biological and radiological arguments with good improvement after medical treatment.

## Clinical presentation

A 77-year-old lady presented to the emergency department with a 4-week history of general deterioration, fever and intermittent abdominal pain located to the upper right quadrant. She denied any vomiting, melena, haematemesis or altered bowel habit. She had no chest pain, dyspnea, cough or urinary symptoms. She had no significant medical history except for cholecystectomy, high blood pressure and osteoarthritis.

On physical examination, she had icterus and tenderness on the right upper quadrant of the abdomen. Murphy’s sign was equivocal. The blood pressure was of 145/77 mm Hg with a normal heart rate and a temperature at 38.9°C.

## Differential diagnosis

All the causes of obstructive jaundice, in particular gallstones and periampullary masses: post pancreatitis collections (pancreatic pseudocyst and infected necrotic collections), periampullary tumors, neoplasm of the head of the pancreas, metastatic lymph node and choledochal cyst.

## Investigations

Laboratory findings revealed a cholestatic hepatitis panel; total bilirubin of 6 mg dl^−1^ with a direct fraction of 3.7 mg dl^−1^, serum aspartate aminotransferase of 234 U l^−1^, alanine aminotransferase of 398 U l^−1^, alkaline phosphatase of 334 U l^−1^, gamma-glutamyl transferase of 399 U l^−1^ and C reactive protein of 40.7 mg dl^−1^. The remaining laboratory values were unremarkable.

## Imaging findings

Abdominal ultrasonography was performed, showing dilation of the intrahepatic bilary tract and the main bilar tract with no evidence of choledocholithiasis

**Figure 1.**
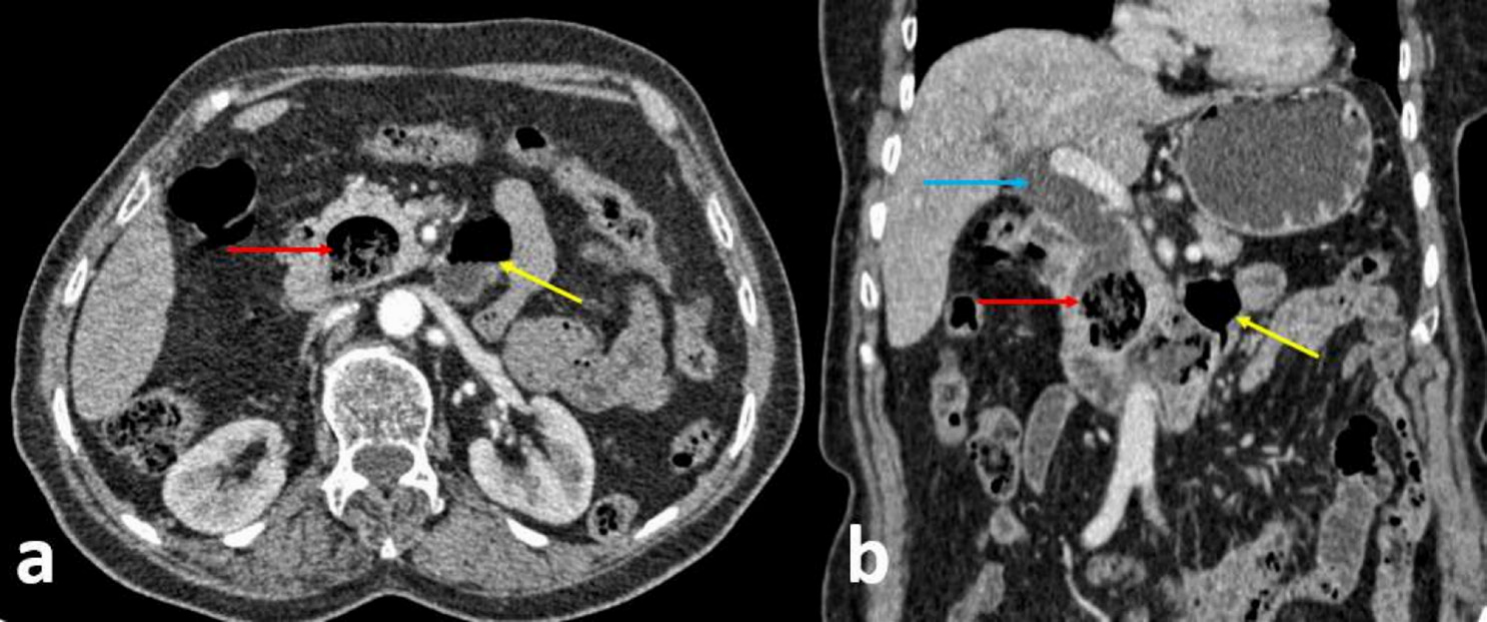
Computed tomography scan of the abdomen with contrast on axial plan (a) and coronal reconstruction (b) showing a 23 mm periampullary duodenal diverticulum in the medial wall of the second portion of the duodenum with containing fluid and air (red arrow) strictly adjacent to the distal portion of the common bile duct (measuring 12 mm in calibre) and causing the dilatation of the bile ducts (blue arrow). Next to the third portion of the duodenum, there was a second diverticulum measuring about 25×21 mm (yellow arrow).

A CT of the abdomen and the pelvic was done and revealed a diverticulum in the medial wall of the second portion of the duodenum with about 23×18 mm, containing fluid and gas with wall thickening, without surrounding inflammatory changes, insinuating itself in the pancreatic head, causing the dilatation of the bile ducts, with no evidence of pancreatic lesions. Next to the third portion of the duodenum, there was a second diverticulum measuring about 25×21 mm. The liver had normal dimensions and no nodular formations were identified. (Figure 1).

**Figure 2.**
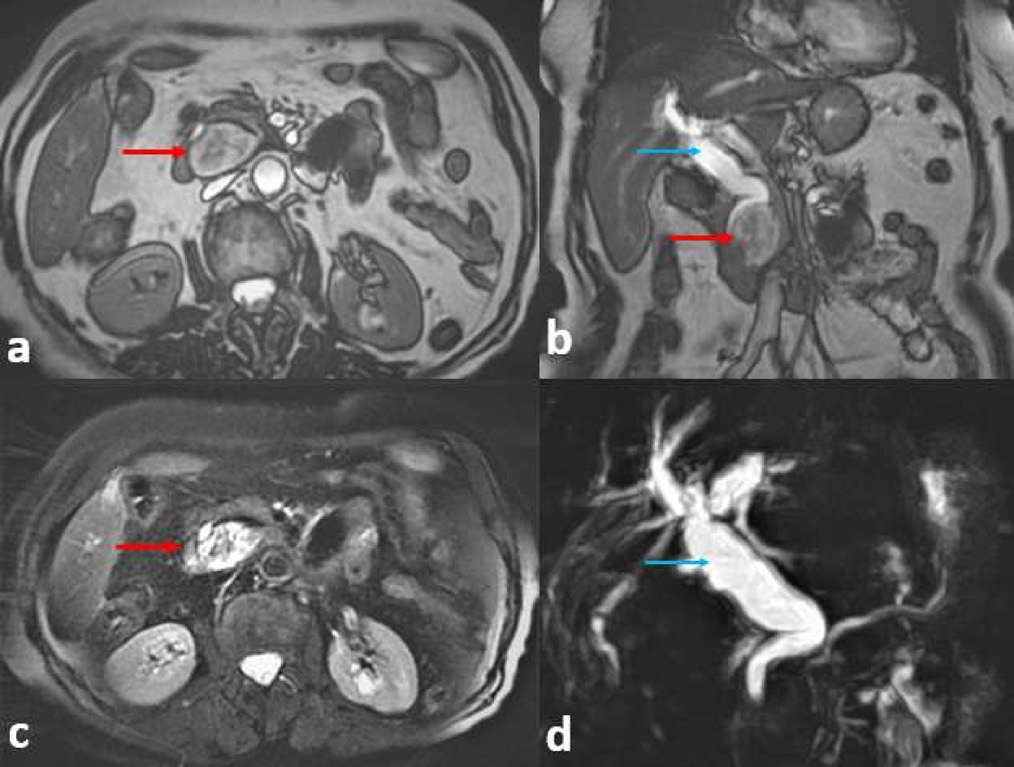
A magnetic resonance cholangiopancreatography imaging with a FIESTA sequence on axial and coronal plan (a, b), T2 FS sequence on axial plan (c) and cholangiographic sequence (d) confirming periampullary duodenal diverticulum (red arrow) and biliary dilation (blue arrow), without pancreatic lesions neither stone evidence.

A magnetic resonance cholangiopancreatography (MRCP) imaging study, which included a cholangiographic sequence, (Figure 2) confirmed the two duodenal diverticulum and biliary dilation, without pancreatic lesions neither stone evidence.

Endoscopic ultrasonography was performed to exclude peri-ampullary tumor. The biopsy did not reveal tumor cells.

A diagnostic of Lemmel’s syndrome was done.

## Treatment, outcome and follow-up

The patient was treated with 14 days course of i.v. wide spectrum antibiotics with an improvement in symptoms and a gradual improvement in markers of cholestasis and inflammation within two weeks. The patient was observed at our outpatient clinic 4 months after discharge, asymptomatic and with normalized laboratory results.

## Discussion

Diverticula are defined as sac-like protrusions of all or part of the bowel wall that can occur anywhere along the gastrointestinal tract. The duodenum is the second most common site for diverticula, after the colon.^1,2^

Duodenal diverticula (DD) are not frequent. Their incidence is 1 to 5% in radiological series and from 11 to 22% in autopsy series. They are most often found in people over 40 years of age. There is no gender predominance.^3^

DDs are asymptomatic in 95% of cases. In about 90%, DDs are solitary. The second part of the duodenum is the most frequent location (about 75%). When DD arise within 2–3 cm from the ampulla of Vater, they are called periampullary, peripapillary, or also paravaterian diverticula. DD are usually discovered incidentally during endoscopic or imaging procedures, but sometimes certain complications may take place. These complications are either non-pancreaticobiliary complications including hemorrhage, perforation, fistula or pancreaticobiliary complications such as recurrent gallbladder or bile duct stones, obstructive jaundice, cholangitis and acute pancreatitis.^4,5^

Lemmel’s syndrome was first described in 1934 by Lemmel. It is defined as obstructive jaundice due to periampullary diverticulum (PAD), in the absence of cholelithiasis or other detectable obstacle. There are only few case reports of this rare syndrome.^1,6^

There are different etiologies regarding the pathogenesis leading to the development of Lemmel syndrome. It may be due to mechanical irritation of the PAD which will cause chronic inflammation of the ampulla, and consequently lead to fibrosis of the papilla. In some cases, PAD can cause sphincter of Oddi dysfunction. Lemmel’s syndrome can be due to mechanical compression of the distal common bile duct or ampulla by PAD.^7^

Pain in the right upper quadrant of the abdomen and jaundice are the two most common symptoms.

In terms of the laboratory workup there is, in the most of cases, a leukocytosis, with an elevation of inflammatory markers, elevated direct and total bilirubin, elevated liver enzymes as in our case direct.^8,9^

Imaging has an important role in the correct and early diagnosis of the disease in order to avoid mismanagement.

Endoscopy examination using a side-view endoscope is the gold standard for a better visualization of PAD. It remains an invasive imaging modality with some complications.

The CT scan and MRCP can be alternative modalities. They allow to visualize the PAD as thin-walled cavitary lesions, but also to study their walls, their contents and look for signs of complications and repercussions on the bilio pancreatic junction.^10,11^

Depending on the underlying symptomology, mechanism and pathogenesis, the modalities of treatment and management may vary.

The conservative management is recommended for asymptomatic patients. Endoscopic extraction, extracorporeal shock wave lithotripsy and diverticulectomy may be considered in case of some proven complications such as biliary obstruction or cholangitis.

The endoscopic sphincterotomy is recommended for chronic papillary fibrosis and sphincter of Oddi dysfunction.^7,12^

## Learning points

- Lemmel syndrome is a rare cause of obstructive jaundice which can mimic several benign and malignant abnormalities in the periampullary region.
- It is essential to always rule out malignant causes of obstructive jaundice by means of CT, MRCP and endoscopy with biopsy.
- The imaging is essential for correct and appropriate diagnosis of Lemmel syndrome and avoid mismanagement.
- The conservative management is recommended for asymptomatic patients.
